# Shoulder Muscle Activation of Novice and Resistance Trained Women during Variations of Dumbbell Press Exercises

**DOI:** 10.1155/2013/612650

**Published:** 2013-05-15

**Authors:** Joshua Luczak, Andy Bosak, Bryan L. Riemann

**Affiliations:** Biodynamics and Human Performance Center, Armstrong Atlantic State University, 11935 Abercorn Street, Savannah, GA 31419, USA

## Abstract

Previous research has compared the effects of trunk inclination angle on muscle activation using barbells and Smith machines in men. Whether similar effects occur with the use of dumbbells or in women remains unknown. The purpose was to compare upper extremity surface electromyographical (EMG) activity between dumbbell bench, incline, and shoulder presses. Dominate arm EMG data were recorded for collegiate-aged female resistance trained individuals (*n* = 12) and novice female resistance trained exercisers (*n* = 12) from which average EMG amplitude for each repetition phase (concentric, eccentric) was computed. No significant differences were found between experienced and novice resistance trained individuals. For the upper trapezius and anterior deltoid muscles, shoulder press activation was significantly greater than incline press which in turn was significantly greater than bench press across both phases. The bench and incline presses promoted significantly greater pectoralis major sternal activation compared to the shoulder press (both phases). While pectoralis major clavicular activation during the incline press eccentric phase was significantly greater than both the bench and shoulder presses, activation during the bench press concentric phase promoted significantly greater activation than the incline press which in turn was significantly greater than the shoulder press. These results provide evidence for selecting exercises in resistance and rehabilitation programs.

## 1. Introduction

Variations of the bench press are commonly used weight training exercises for strength and power development of the muscles in and around the chest and shoulder areas. For example, although the bench press is most often performed with a standard barbell and stable flat bench, dumbbells and machines can also be used as sources of resistance along with unstable surfaces (i.e., cushions and Swiss balls) to produce similar press actions with varying intensity and stabilization demands. Understanding the mechanical demands imposed by each bench press variation assists with matching the patient/client needs with the specific characteristics of each exercise he/she completes. 

Changing the angle of trunk inclination during variations of bench pressing exercises changes the line of action of the resistance relative to the trunk and, in turn, changes the direction of the movement force provided by the shoulder muscles. Based on the arrangement of the shoulder muscles, accommodation to these changes in bench inclination is thought to promote maximal activation of the muscles active during variations of bench press exercise involving sets of varying inclinations. The pectoralis major has two different heads, sternocostal and clavicular, and, collectively, this group provides adduction and medial rotation at the shoulder joint [1]. Additionally, the sternocostal head is a shoulder horizontal adductor, which can be helpful during the horizontal bench press. Previous research has shown the sternocostal head to be most active during the decline or horizontal bench [2–4]. The clavicular head can also be a shoulder flexor [1]. The shoulder flexion movement is more apparent in the incline press, and increased activation of the clavicular head has been documented in prior studies [2, 5]. The anterior deltoid is responsible for flexion, abduction, and medial rotation about the shoulder joint [1]. Abduction of the shoulder is a primary movement during the military shoulder press, which makes that exercise good for exercising the anterior deltoid, and previous studies have confirmed the use of the anterior deltoid during this movement [2, 6]. All of the above-mentioned studies used surface electromyography (EMG) for analysis of muscle activation. However, a muscle that has not been considered for analysis is the upper trapezius. This specific muscle is responsible for the elevation of the shoulder joint [1], which combines with shoulder abduction during pressing exercises. 

Another consideration in the bench press is the phase of the contraction. Eccentric and concentric muscle actions occur throughout the full range of the exercise. Previous research offers varying results concerning activation differences between the concentric and eccentric phases of a bench press exercise. While muscle activity did not differ between the two phases when using relatively high resistance like 80% 1 RM [6] or a true 1 RM [4, 7], activation during the concentric phase was revealed to be higher compared to the eccentric phase at relatively low resistances like 60% 1 RM [8] and 70% 1 RM [3]. These studies either did not use dumbbells or were focusing their investigation on core muscle activation during a press exercise on a Swiss ball. 

Definitions of weight “trained” and “untrained” individuals have varied throughout studies. In previous work utilizing EMG during bench press exercises, “trained” subjects have been defined as those who have regularly performed the bench press for at least 1 year [9] or performed the bench press twice per week for 6 months [10]. In addition, “untrained” subjects have been defined as not having regularly performed the bench press for 2 years [9] or just not meeting the criteria to be considered “trained” [10]. No differences in muscle activity have been observed between experience levels, despite the knowledge that improvements via strength training include neuromuscular efficiency [11]. Because what governs the status of being considered “trained” has varied in previous studies, the issue of activation differences between novice and experienced resistance trained exercisers requires further research. 

Prior studies have considered muscle activation during bench press exercise using barbell [2–5, 8, 12–14], Smith [4], dumbbells [4, 6, 12, 14], and unstable versus stable surfaces [6–8, 12]. A few studies have compared muscle activation among press exercises conducted with varying inclinations [2, 5]. Although one study [6] included bench and shoulder press exercises with dumbbells, there were no direct muscle activation comparisons made between the two variations. Similar to the concept of promoting more stabilizer activity with free weights compared to machines, dumbbells should further increase the stabilization demands [4]. Across the whole exercise (both eccentric and concentric phases), Saeterbakken et al. [4] reported significantly greater biceps and less triceps muscle activity using dumbbells for the bench press compared to using a barbell or Smith machine. They reported no differences for anterior deltoid and pectoralis major muscles. Based on their results, it is plausible that the activation differences previously reported between varying angles of inclination during press exercises with barbells and Smith machines may be less when performed with dumbbells. Additionally, because the majority of the previous research has only included men, little is known about muscle activation during press exercises in women. Therefore, the purpose of this study was to examine muscle activity of the anterior deltoid, pectoralis major (clavicular and sternal portions), and upper trapezius during horizontal, incline, and shoulder presses using dumbbells in young adult women. In addition, because the effects of weight training experience on muscle activity during press exercises remain inconclusive, comparisons were also made between experienced and novice resistance trained exercisers. We hypothesized that varying angles of inclination would have a potent effect on the upper trapezius but marginal effects on the anterior deltoid and pectoralis major muscles. In addition, we hypothesized that the novice resistance trained individuals would have higher muscle activation than experienced lifters, and the concentric phase would have higher activation than the eccentric phase. 

## 2. Methods

### 2.1. Subjects

Twenty-four healthy college-aged recreational female athletes were recruited as subjects (Table 1). Exclusion criteria included subjects with shoulder, elbow, or wrist injuries within the past six months or who were athletes in a sport that emphasized the use of one arm over the other (i.e., tennis, softball, and volleyball). Subjects were divided into two groups, twelve per group, based on their experience with upper body weight training. One group was identified as experienced resistance trained exercisers by regularly participating in upper body resistance exercise at least 1–3 times per week for the last six months. The second group was comprised of novice resistance trained females who did not regularly participate in upper body resistance training exercises but instead were physically active in cardiovascular exercise at least 1–3 times per week. Before participation, the researchers gave a full overview of the study's procedures to the participants. The participants conveyed their understanding about the requirements of participating in the study by signing an informed consent form approved by the Armstrong Atlantic State University Institutional Review Board. Once informed consent was attained, all participants completed an activity history and comprehensive medical screening questionnaire to verify their eligibility.

### 2.2. Experimental Design

This experiment utilized a 2-way repeated measures design. Exercises were administered in a between-subjects counterbalanced order. The objective was to measure muscle activity in the anterior deltoid, pectoralis major (clavicular and sternal portions), and upper trapezius muscles by way of surface electromyography data collection during flat bench (0° trunk inclination), incline bench (45°), and shoulder (85°) presses and then compare the muscle activation data between the novice and experienced groups as well as between the different exercises during the concentric and eccentric phases.

### 2.3. Procedure

Participants were instructed in proper technique for the three exercises as outlined by Earle and Baechle [11] with 4.5 kg dumbbells in each hand at their own pace. Emphasis was placed on the participants completing each repetition in a controlled manner. Participants completed at least three practice trials of each exercise prior to the trials completed when data collection occurred. During the practice trials, participants were given further verbal cues to correct form. Completing the repetitions with full elbow extension and the hands positioned over the shoulder joints was encouraged. Once acceptable form was obtained, participants completed one set of five repetitions each of the flat bench (BP), 45° incline (IP), and 85° shoulder (SP) presses using 4.5 kg dumbbells during which data collection occurred. The order of the exercises was completed according to the counterbalanced protocol that each subject was assigned. Initiation of each set of repetitions was self-initiated and three minutes of rest were given between sets. 

### 2.4. Instrumentation and Data Collection

Electromyographic data were collected from the participant's dominant arm (preferred arm used to throw a ball) using rectangular-shaped bipolar (1 cm interelectrode distance) (DelSys DE-2.1, DelSys, Inc, Boston, MA, USA) 99.9% Ag surface electrodes. Before the placement of the electrodes, electrode sites were shaved, abraded with sandpaper, and cleaned with alcohol at each site. Double-sided tape was used to attach the electrode to the skin, and athletic tape was placed over the electrode to ensure it would stay in place. Electrode placement for the upper trapezius and anterior deltoid followed the recommendations of The Surface Electromyography for the Non-Invasive Assessment of Muscles Project [15]. For the upper trapezius, electrodes were placed at 50% of the distance between the acromion process and the spine of the seventh cervical vertebra. For the anterior deltoid, electrodes were placed at one finger width distal and anterior to the acromion. Placement of the electrodes for the pectoralis major sites followed different recommendations [15, 16]. For the clavicular portion of the pectoralis major, electrodes were placed four finger widths below the medial clavicle just medial to anterior axillary border, and for the sternal portion, electrodes were placed four finger widths below the sternoclavicular joint. A ground electrode was placed on the olecranon process of the elbow where no electrical activity would occur. 

The Bagnoli-8 System (Delsys, Inc, Boston, MA, USA), raw electromyographical (EMG) data, was used to acquire, filter (20 to 450 Hz), and amplify EMG data with a minimum common mode rejection ratio >84 dB and input impedance >10 K*Ω*. To ensure accurate placement of the electrodes, manual muscle tests were performed at each muscle following recommendations made by Daniels and Worthington [17] and viewed by the oscilloscope in the MotionMonitor acquisition software package (Innovative Sports Training Inc., Chicago, IL, USA). Adjustments to amplifier gains (100, 1 k, and 10 k) were made as needed to maximize signal resolution. Data were then analog to digital converted (1000 Hz) (ComputerBoards PCM16S/12, ComputerBoards, Inc., Middleboro, MA, USA, USA) and stored on a desktop computer using the MotionMonitor data acquisition software (Innovative Sports Training, Inc., Chicago, IL, USA). Additionally, a digital video camera (Sony Handycam DCR-HC52), synchronized with the EMG data collection, captured all repetitions and was used to identify phases of the exercises. Before completing the trials, EMG data were recorded for ten seconds with the participant sitting still and erect to establish zero baseline muscle activity. During the trials, activity recording was saved for 25 seconds after a 0.5 V impulse was sent to the computer to initiate the process.

### 2.5. Data Reduction

Using the recorded video data, the corresponding frame numbers were selected for each subject during each repetition of each exercise to identify the beginnings and ends of the eccentric and concentric phases for each repetition. The beginning and end of a repetition was defined by full elbow extension. When the elbows began to flex and the weight moved toward the chest, the beginning of the eccentric phase was identified. The end of the eccentric phase and beginning of the concentric phase were identified when the weight reached a point where its direction changed from inferior to superior movement. All further EMG data reduction was conducted using the MATLAB-based scripts (MathWorks, Inc., Natick, MA, USA). Adjustments were made according to the gains for each muscle and resting baseline EMG activity was subtracted. After the raw EMG data was rectified and low pass filtered (10 Hz), ensemble averages were calculated separately for each repetition of each exercise by converting the time of repetition into percentages and then averaging the muscle activation at each percentage across the entire repetition. Amplitude normalization for all exercises was conducted using the mean ensemble average [18] from the flat bench press repetitions. The average activity from the ensemble averages for the eccentric and concentric phases of each exercise were computed and used for data analysis. Additionally, repetition, eccentric phase, and concentric phase times were computed using the frame number selected and averaged across the five repetitions.

### 2.6. Data Analysis

All statistical analyses were conducted using SPSS Statistics Release 19 (IBM, Inc., Armonk, NY, USA). Three separate 1-way repeated measures analyses of variation (ANOVA) were used to compare repetition, eccentric phase, and concentric phase times between the three exercises. Four separate 3-way (group × exercise × phase) repeated measures ANOVA were used for each of the four muscles. Before conducting the ANOVAs, normality was verified through an analysis of the Q-Q plots, while sphericity was verified through Mauchly's test. If sphericity was rejected, the Huynh-Feldt correction was used. Where significant interactions were identified, simple main effect post hoc tests with Bonferroni corrections were used. To provide indications about the size of the pairwise differences, 95% confidence intervals (95% CI_diff_) were computed. Statistical significance was considered at *P* ≤ 0.05. 

## 3. Results

All 24 subjects were able to complete five repetitions for each exercise at the three different trunk inclinations. There were no significant differences (*P* > 0.05) between experienced and novice weight lifters or between the three exercises for repetition, eccentric phase, and concentric phase times (Table 2).

### 3.1. Upper Trapezius

No significant differences between the experienced and novice resistance trained females were revealed in the exercise × group (*P* = 0.625), phase × group (*P* = 0.504), or exercise × phase × group (*P* = 0.863) interactions; however, a significant exercise × phase interaction (*F*_1.4,  31.5_ = 20.4, *P* < 0.001) was identified (Table 3). For the eccentric phase, significant differences in muscle activation were identified between the three exercises: BP < IP (*P* < 0.001, 95%  CI_diff_: 117–301%), BP < SP (*P* < 0.001, 95%  CI_diff_: 109–617%), and IP < SP (*P* < 0.001, 95% CI_diff_: 471–821%). Significant differences between the three exercises were also identified during the eccentric phase: BP < IP (*P* < 0.001, 95% CI_diff_: 163–380%), BP < SP (*P* < 0.001, 95% CI_diff_: 774–1348%), and IP < SP (*P* < 0.001, 95% CI_diff_: 578–1001%). During the bench (*P* = 0.009, 95% CI_diff_: 2–12%), incline (*P* = 0.002, 95% CI_diff_: 28–111%), and shoulder (*P* < 0.001, 95% CI_diff_: 126–300%) presses, muscle activation during the concentric phase was significantly higher than during the eccentric phase.

### 3.2. Anterior Deltoid

No significant differences between the experienced and novice resistance trained individuals were revealed in the exercise × group (*P* = 0.756), phase × group (*P* = 0.747), or exercise × phase × group (*P* = 0.054) interactions; however, a significant exercise × phase interaction (*F*_1.3,  29.1_ = 4.8, *P* = 0.028) was identified (Table 3). For the eccentric phase, significant differences in muscle activation were identified as follows: BP < IP (*P* < 0.001, 95% CI_diff_: 61–110%), BP <SP (*P* < 0.001, 95% CI_diff_: 137–231%), and IP < SP (*P* < 0.001, 95% CI_diff_: 64–134%). Significant differences between the three exercises were also identified during the concentric phase as follows: BP < IP (*P* < 0.001, 95% CI_diff_: 100–163%), BP < SP (*P* < 0.001, 95% CI_diff_: 137–306%), and IP < SP (*P* = 0.003, 95% CI_diff_: 29–152%). During the bench (*P* < 0.001, 95% CI_diff_: 53–76%), incline (*P* < 0.001, 95% CI_diff_: 90–132%), and shoulder (*P* < 0.001, 95% CI_diff_: 59–145%) presses, activation during the concentric phase was significantly higher than that during the eccentric phase. 

### 3.3. Pectoralis Major: Sternocostal Head

No significant differences between the experienced and novice resistance trained exercisers were revealed in the exercise × group (*P* = 0.532), phase × group (*P* = 0.353), or exercise × phase × group (*P* = 0.830) comparisons; however, a significant exercise × phase interaction (*F*_1.7,   37.9_ = 50.6, *P* < 0.001) was identified (Table 3). For the eccentric phase, significant differences in muscle activation were revealed during the eccentric phase between the SP and BP (SP < BP, *P* < 0.001, 95% CI_diff_: 69–105%) and IP (SP < IP, *P* < 0.001, 95% CI_diff_: 23–46%), while no significant difference (*P* = 0.779) occurred between BP and IP. Similarly, significant differences in muscle activation were identified between the SP and BP (SP < BP, *P* < 0.001, 95% CI_diff_: 69–105%) and SP (SP < IP, *P* < 0.001, 95% CI_diff_: 60–89%), with no significant difference (*P* = 0.229) between the BP and IP. During the BP (*P* < 0.001, 95% CI_diff_: 40–67%) and IP (*P* < 0.001, 95% CI_diff_: 35–54%), activation during the concentric phase was higher than during the eccentric phase. There was no significant difference between the concentric and eccentric phases for the SP (*P* = 0.544).

### 3.4. Pectoralis Major: Clavicular Head

No significant differences between the experienced and novice resistance trained individuals were revealed in the exercise × group (*P* = 0.244), phase × group (*P* = 0.410), or exercise × phase × group (*P* = 0.364) interactions; however, a significant within-subjects effect was found in the exercise × phase interaction (*F*_2, 44_ = 43.2, *P* < 0.001) and was identified (Table 3). For the eccentric phase, significant differences in muscle activation were identified between the IP and BP (BP < IP, *P* < 0.001, 95% CI_diff_: 18–55%) and SP (SP < IP, *P* = 0.002, 95% CI_diff_: 11–50%); however, there was no significant differences between BP and SP (*P* = 1.000). For the concentric phase, significant differences were revealed between all three exercises: BP < IP (*P* = 0.001, 95% CI_diff_: 18–71%), SP < BP (*P* = 0.005, 95% CI_diff_: 11–69%), and SP < IP (*P* < 0.001, 95% CI_diff_: 61–108%). During the BP (*P* < 0.001, 95% CI_diff_: 47–73%), IP (*P* < 0.001, 95% CI_diff_: 52–85%), and SP (*P* = 0.001, 95% CI_diff_: .7–21%), activation during the concentric phase was significantly higher than during the eccentric phase. 

## 4. Discussion

Because weight training individuals often seek to get the most training effect from their workouts, it is important to understand which exercise and training medium would provide the greatest activation of a targeted muscle mass. Thus, the purpose of this study was to examine muscle activity of the anterior deltoid, pectoralis major (clavicular and sternal portions), and upper trapezius during horizontal, incline, and shoulder presses with dumbbells. The results of this study largely confirm previous research related to performing these exercises with barbells. Specifically, the bench and incline presses produced the greatest activation for the two portions of the pectoralis major muscle, while the shoulder press elicited the greatest activation for the anterior deltoid and upper trapezius muscles.

Additionally, because it would intuitively seem that differences in muscle activation may exist between persons with varying weight training experience, we sought to consider these groups independently. The comparisons between novice and experienced lifters showed no significant difference in muscle activation for all of the muscles considered. This coincides with results from other studies which found no differences between groups of different training levels. Specifically, studies involving women tested at 60% and 80% 1 repetition maximum (RM) intensities [9] and men tested at 70% and 90% 1 RM intensities in men [10] failed to demonstrate differences in muscle activation based upon experience level. Despite the current study using a standardized weight for each subject rather than intensities relative to 1 RM and utilizing dumbbells instead of barbells, no differences in muscle activation were observed between the novice and experienced resistance trained individuals. While it has been suggested that neuromuscular efficiency is improved initially in strength training [11, 13], and since a standardized load was used in our study, the present study design did not allow the determination of neuromuscular efficiency between the experimental groups. Our definition of a trained subject was having regularly participated in upper body resistance exercise at least 1–3 times per week for the last 6 months or more. Our novice group regularly participated in cardiovascular exercise but had not engaged in upper body resistance exercise in the prior six months. It is important to note that we relied on self-reported exercise data to classify the subjects into the two groups. Perhaps if we had our subjects complete more repetitions or use a greater resistance, we may have identified experience-related differences. Another explanation for the lack of group differences may have been related to us only assessing prime mover muscles. Hence, it is possible that there are activation differences in muscles we did not assess, such as the rotator cuff muscles. Future research should consider evaluating those muscles associated with stabilization roles of the shoulder complex.

The results for the upper trapezius indicate higher muscle activity as the angle of trunk inclination increased. Activation during the shoulder press was higher than during the incline press, which in turn was higher compared to the bench press. Because the upper trapezius is the primary scapular elevator/upward rotator, these results are not surprising given the demands for upward scapular rotation during each of these exercises. Extensive literature searching failed to reveal any previous studies considering upper trapezius activation during press exercises. 

Results for the anterior deltoid are comparable to the upper trapezius with respect to higher muscle activation as the angle of trunk inclination increases. This supports previous findings that indicated a greater anterior deltoid activation during higher angles of inclination [5, 7]. Similar to the upper trap, the explanation for these differences is likely related to its movement responsibility as a shoulder abductor and flexor by being more active during exercises that require more shoulder flexion to complete [5].

Conversely, the clavicular portion of the pectoralis major exhibited similar activity level differences as previous studies by being more active during the incline press compared to the bench press [2, 5] and shoulder press [2]. In addition, consistent with Trebs et al.'s study [5], activation during the concentric phase of the incline press was significantly greater than the bench press. Despite similar angles of inclination for the bench and incline presses, our results regarding the sternal portion of pectoralis major were different than previous research [5] which reported bench press activation to be significantly greater than incline press. In our results, no significant difference between the incline and horizontal presses was observed, which is also consistent with Barnett and kippers' study [2]. Thus, it would largely appear that similar trends of pectoralis major activation occur across the three different angles of inclination regardless if barbell or dumbbells are used.

As far as comparisons of muscle activation during the concentric and eccentric phases, our results support the finding of previous studies that suggested at lesser submaximal testing [3, 8], activation is higher during the concentric phase versus the eccentric phase. This may be due to there not being a need for as much stabilization of the weight during the descent versus what is required during the completion of a higher %RM lift. However, other studies that used a higher submaximal resistance or a 1 RM [6, 7] did not see a difference in activation between the eccentric and concentric phases.

As previously discussed, the majority of research examining muscle activation during the various inclinations of press exercises has been conducted using men subjects. Also unique to the current study was the use of dumbbells as a source of resistance rather than barbell or Smith machine. The results of our study largely extend the results of the earlier studies to women and the use of dumbbells. It is important to note, however, that we used minimal resistance. Whether similar results would have been attained with greater resistance is unknown and should be examined by future research. Furthermore, once the practice trials were completed, we did not assess or attempt to influence the patterns of movement used to complete the three bench press variations. Examining the patters of movement throughout the upper extremity between the three bench press variations is recommended for future research. Finally, it is important to note that our electromyographical data was normalized to the mean muscle activity demonstrated across the whole bench press movement. Amplitude normalization was only needed in the current study because of comparisons being conducted between two groups of participants. Because the bench press is the most commonly used press exercise, this was chosen as the normalization standard. By using this standard, the values reported during the concentric and eccentric phases of each press variation can be readily interpreted to what occurs across the whole bench press movement.

## 5. Conclusion

The current results provide evidence for selecting dumbbell press variations in resistance and rehabilitation programs. While there were no differences related to resistance training experience, there were differences revealed between the three bench presses. Of the three bench presses, the shoulder press best targeted the upper trapezius and anterior deltoid. The incline press promoted the greatest activation of both parts of the pectoralis major. While these results largely extend the previous research which used barbells and Smith machines compared to dumbbells, the results of this study can only be generalized to healthy, college-aged women. Future research investigating dumbbell press exercise should consider differences in shoulder girdle stabilizers as well as greater exercise intensity. 

## Figures and Tables

**Table 1:** Subject demographics (mean ± standard deviation).

Group	*N*	Mass (kg)	Height (m)	Age
Novice	12	64.1 ± 10.1	1.69 ± 0.07	23.0 ± 2.8
Experienced	12	64.6 ± 8.0	1.69 ± 0.04	22.6 ± 1.7

**Table 2:** Mean ± standard deviation for the three time-related variables across all subjects (*n* = 24). There were no significant differences between the dumbbell press exercises.

	Bench press	Incline press	Shoulder press
Eccentric phase time (s)	1.28 ± .16	1.32 ± .36	1.30 ± .31
Concentric phase time (s)	1.22 ± .21	1.16 ± .34	1.21 ± .27
Total repetition time (s)	2.51 ± .36	2.48 ± .42	2.51 ± .54

**Table 3:** Mean ± standard deviation activation amplitude across all subjects (*n* = 24) for each muscle by exercise phase expressed as a percentage of the mean ensemble averages of the bench press (entire repetition). With the exception of the pectoralis major sternal, concentric activation amplitude was significantly greater than eccentric activation amplitude within each muscle.

	Concentric			Eccentric		
	Bench press	Incline press	Shoulder press	Bench press	Incline press	Shoulder press
Upper trapezius	103.5 ± 6.4	374.0 ± 201.7 ∗	1164.7 ± 533.2^†^	96.6 ± 6.0	305.6 ± 169.4 ∗	951.7 ± 443.3^†^
Anterior deltoid	132.8 ± 13.5	264.1 ± 59.4 ∗	354.5 ± 159.9^†^	68.1 ± 14.6	164.4 ± 49.0 ∗	252.3 ± 86.8^†^
Pectoralis major sternal	127.2 ± 16.0^‡^	114.3 ± 25.1^‡^	40.0 ± 21.6	73.5 ± 15.9^‡^	70.2 ± 22.8^‡^	38.7 ± 26.5
Pectoralis major clavicular	130.5 ± 14.8^‡^	175.0 ± 50.1^∗‡^	90.8 ± 47.3	70.6 ± 14.6	106.8 ± 42.3^∗‡^	76.7 ± 46.0

^*^Significantly greater than bench press.

^†^Significantly greater than bench press and incline press.

^‡^Significantly greater than shoulder press.
